# Herb Network Analysis for a Famous TCM Doctor's Prescriptions on Treatment of Rheumatoid Arthritis

**DOI:** 10.1155/2015/451319

**Published:** 2015-04-23

**Authors:** Yan Li, Rui Li, Zibo Ouyang, Shao Li

**Affiliations:** ^1^Yijishan Hospital, Wannan Medical College, Wuhu 241001, China; ^2^MOE Key Lab of Bioinformatics, Bioinformatics Division, TNLIST, Department of Automation, Tsinghua University, Beijing 100084, China

## Abstract

Traditional Chinese Medicine (TCM) doctors always prescribe various herbal formulae tailored to individual patients. However, there is still a lack of appropriate methods to study the rule and potential biological basis underlying the numerous prescriptions. Here we developed an Herb-Compound-Target-Disease coherent network approach to analyze 871 herbal prescriptions from a TCM master, Mr. Ji-Ren Li, in his clinical practice on treatment of rheumatoid arthritis (RA). The core herb networks were extracted from Mr. Li's prescriptions. Then, we predicted target profiles of compounds in core herb networks and calculated potential synergistic activities among them. We further found that the target sets of core herbs overlapped significantly with the RA related biological processes and pathways. Moreover, we detected a possible connection between the prescribed herbs with different properties such as Cold and Hot and the Western drugs with different actions such as immunomodulatory and hormone regulation on treatment of RA. In summary, we explored a new application of TCM network pharmacology on the analysis of TCM prescriptions and detected the networked core herbs, their potential synergistic and biological activities, and possible connections with drugs. This work offers a novel way to understand TCM prescriptions in clinical practice.

## 1. Background

Traditional Chinese Medicine (TCM) is one of the most treasured cultural heritages in China and is also an inseparable part of current medical systems. Herbal formula (*Fang-Ji* in Mandarin) that consists of two or more medicinal herbs is the major form in TCM clinical practice. Nowadays TCM herbal formula has been well known to be effective for the control of many complex diseases, for example, rheumatoid arthritis (RA), a common autoimmune disease that leads to a chronic and systemic inflammatory disorder [1, 2]. Rheumatoid arthritis is manifested by synovial inflammation, morning stiffness and pain, and may affect many tissues and organs in human body, especially causing joint deformity. Rheumatoid arthritis is a complex disease with limited treatment options. Without regular treatment, many patients tend to suffer from high probability of disability. TCM has been regarded as an important strategy to inhibit RA development and improve the life quality of RA patients [3]. Actually, the treatment of RA in TCM can be traced up to more than 3000 years ago. RA falls in the scope of the TCM therapeutic concept “*Bi ZHENG*.” The etiologies, syndrome classification, and treatment of* Bi ZHENG* had been recorded in the Yellow Emperor's Internal Classic, one of the earliest treatises in TCM. Subsequently, TCM practitioners have accumulated rich clinical experience and numbers of herbal formulae for treating patients with rheumatoid arthritis. As one of the first 30 “Grand Masters of TCM” selected by Chinese government, Mr. Ji-Ren Li at Yijishan Hospital, Wannan Medical College, is a highly regarded Chinese medicine practitioner who is skilled in the treatment of RA [4, 5]. Mr. Li has accumulated numerous herbal prescriptions to cure RA patients and early action needs to be taken to understand the professional experience embedded in such valuable herbal prescriptions.

However, quite different from the clinical practice of the Western medicine, herbal formulae are always prescribed by TCM practitioners specifically tailored to individual patients. TCM doctors treat patients mainly based on syndrome differentiation. For different patients, various herbs are grouped and different formulae are prescribed. This tradition and feature make TCM a kind of personalized medicine and cause great difficulty in designing standard protocols such as the randomized controlled trial in TCM studies. The lack of appropriate methods to analyze the rule as well as scientific basis for TCM-doctor-prescribed herbal formulae is therefore one of the major frustrations to find clinical evidence of TCM. How to study the characteristics and prescription rule of such flexible TCM formulae is still a great challenge and requires widely interdisciplinary efforts.

Thanks to the rapid progress of bioinformatics especially complex network theory and technologies, recently, the network approach has become a new avenue and powerful tool to handle intricate problems such as those in TCM. We have been making efforts to build a TCM network pharmacology approach over ten years and established a set of network-based methods for TCM study [6–9]. For example, to reveal the combination rule and the biological basis of herbal formulae, we proposed a global network analysis framework consisting of herb/compound network, biological network (gene, protein, or target network), and disease network. Several methods are also created such as a distance-based mutual information model (DMIM) that can identify herb relationships from numerous herbal formulae. The good performance of the DMIM method to uncover the herbal combination rules has been illustrated in both 3865 collaterals-related herbal formulae and a classic* Liu-Wei-Di-Huang* formula [6]. Furthermore, we proposed a novel concept of “network target” [7], which treats a disease-specific molecular network as a therapeutic target to help design suitable medical treatments. Several methods for the network target analysis especially the disease gene prediction, target prediction for drugs or herbal compounds [8], the network comodule analysis for drug discovery, and the network-based prioritization of synergistic combinations [7] are developed subsequently. These methods have been applied in finding bioactive compounds and revealing the mechanism of action for herbal formulae including* Liu-Wei-Di-Huang* pill [9]. Now the network-based method in the context of TCM network pharmacology has become a burgeoning field in the modern studies of TCM [10–12]. We believe the network-based strategy and approaches can also provide good solutions for the herbal prescription analysis.

In this work, 871 herbal prescriptions from Mr. Ji-Ren Li's clinical treatment on patients with rheumatoid arthritis were analyzed from a network point of view. TCM network pharmacology methods were employed to detect the common rules and potential biological basis of these herbal prescriptions. This work is promising as the results nicely show the rationality and validity of Mr. Li's herbal prescriptions. Moreover, this work provides a new route to interpret the professional experience embedded in numerous and precious TCM herbal prescriptions.

## 2. Methods

### 2.1. Herbal Formulae and Herbal Compounds

A total of 871 herbal prescriptions from Mr. Ji-Ren Li in the individually clinical treatment of RA patients from 2005 to 2013 were used in this work. The 871 herbal prescriptions were normalized by substituting the polysemes, synonyms, and acronyms of the herbs in the dataset and resulted in a standardized Herb Name list. Each formula contains 15 to 29 herbs and the average number is 18. Compounds of herbs were gathered from the HerbBioMap database (China Copyright of Computer Software, 2011SR076502). The HerbBioMap database contains 10806 compounds for 539 herbs up to now.

### 2.2. Multilayer Coherent Network Analysis for Herbal Prescriptions

**Figure 1:**
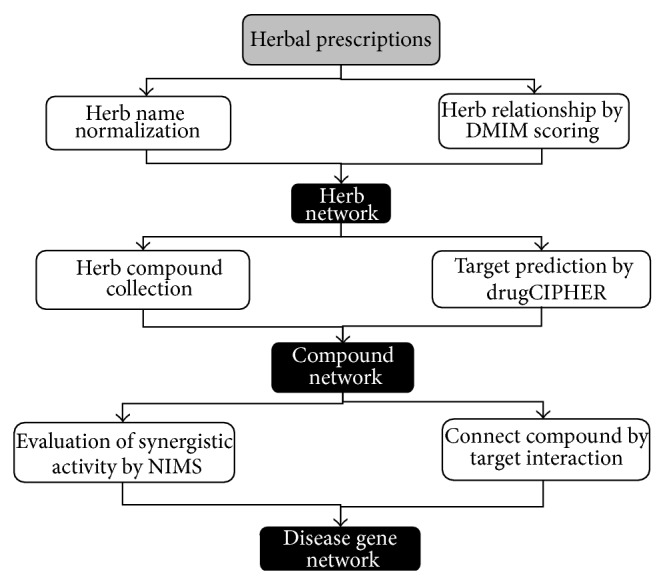
The Herb-Compound-Target-Disease coherent network analysis framework for studying herbal prescriptions on the treatment of certain diseases.

As shown in Figure 1, we proposed an Herb-Compound-Target-Disease coherent network analysis framework to study the prescription rules and potential biological basis for herbal prescriptions. The principle steps are summarized as follows.

*Herb Network Construction*. We created a matrix of 871 dimensions to represent all collected herbal formulae and then calculated the frequency of every 343 herbs in all formulae and gave a normalized frequency (NF), NF = AF/∑_*n*=1_^*N*^AF_1_, where AF is accumulated frequency and *N* denotes 343-herb species number. Next, most frequent herbs in 871 herbal prescriptions were selected and subjected to the DMIM (distance-based mutual information model) analysis, an algorithm for identifying herb relationships among different formulae and uncovering the combinational rules of herbs in a network manner [6]. The DMIM model is an integrated scoring system combining the mutual information (MI) entropy characteristics and the “between-herb distance” (*d*) between herbs by calculating score(*x*, *y*) = MI(*x*, *y*)/*d*(*x*, *y*), which describes the tendency of herb *x* and herb *y* to form an herb pair. More importantly, the DMIM model extracts information from the organization of* Jun-Chen-Zuo-Shi* (Master-Adviser-Soldier-Guide) herbs in an herbal formula, based on which a new parameter “between-herb distance” was created that means the further the distance between two herbs in a formula is, the less likely they are to be relevant to one another. Thus, DMIM is suitable to retrieve herb combinational rules, achieving a good balance among the herb's frequency, independence, and distance in herbal formulae [6].

*Target Prediction for Herbal Compounds and Herbs*. In the present study, drugCIPHER, a method we developed previously [8], is used to predict the target profile for all available compounds collected from each herb in the core herb networks. drugCIPHER is a regression model that can predict the links between drugs and target proteins in a genome-wide scale by correlating the drug chemical information and protein-protein interaction (PPI) from a heterogeneous network. By integrating and making full use of all current FDA-approved drug structures, drug-target interactions, and human protein-protein interactions, drugCIPHER can output a target profile for any given compound with known structure that is calculated from the correlation between the query ingredient's structure similarity vector in the drug space and the target-related gene's closeness vector in the target space. As the top 100 targets predicted by drugCIPHER can reach the high prediction accuracy (77.3%) [8], here, the top 100 target profiles predicted for each compound were selected, and the target profiles of all collected compounds within a herb were combined to form integrative target profiles of this herb. The target prediction procedure was conducted for herbs present in the core herb networks or involved in the following studies.

*Evaluation of Synergistic Actions for Herb Network*. To identify the potential synergistic effects of herb combinations in the core herb network at the molecular level and in a high throughput way, we used the network-based identification of multicomponent synergy (NIMS) method [7], a novel approach to access the synergistic strength of any combination of herbal compounds by determining their target interactions in the protein-protein network. The NIMS algorithm consists of two components: topology score and agent score. The topology score is generated from the topological features of the background network related to disease condition and drug actions, while the agent score is based on the similarity of agent phenotypes. Two graph-based measures, betweenness and closeness, were used to capture the associations among targets, and the other measure, PageRank, to verify the node importance [7]. The resulted synergy score is used to prioritize the synergistic effect of combinations of agents including herbs or herbal compounds.

*Gene Ontology (GO) and Pathway Analysis for Herb Targets and RA Genes*. The targets of each compound in the top herbs were predicted using drugCIPHER. For each compound of the top herbs, the top 100 proteins were treated as targets for the given compound. The targets hit by more than five compounds within an herb were considered herb targets. Top 15 frequent herbs' targets were subjected to the gene ontology [13] and pathway enrichment analysis. One the other hand, the 129 RA-related genes with a direct mechanism relationship to rheumatoid arthritis were collected from the CTD database [14]. For functional analysis, we used the “Set Analyzer” tool at the CTD site (http://ctdbase.org/tools/analyzer.go) to analyze the enriched GO terms as well as KEGG pathways for herb targets and RA genes, respectively, with corrected *P* value less than 0.05. We also mapped the RA genes and herb targets in the enriched KEGG pathway network.

*Compound Network Analysis for Anti-RA Herbs and Drugs*. For comparison of the herbal prescriptions and the Western anti-RA drugs, we collected the commonly used anti-RA drugs and their targets from the DrugBank database [15]. Nine immunomodulatory drugs and 7 hormones were selected. The immunomodulatory drugs include methotrexate, leflunomide, ciclosporin, thalidomide, cyclophosphamide, azathioprine, mycophenolate mofetil, tacrolimus, and cyclosporine. Hormones include dexamethasone, hydrocortisone, cortisone, triamcinolone, prednisolone, prednisone, and methylprednisolone. Based on the targets of each compound contained by the top 50 frequent herbs, the closeness of herb compound's targets and targets of immunomodulatory drugs and hormones on the RA-related gene network that connected by PPI interactions (HPRD, Release 7) [16] were measured. The compound networks are then formed in which two compounds (or drugs) will be linked if their targets are overlapped or directly connected in the network. Here we paid more attention on the relationship between herbal compounds with Cold/Hot property and two types of RA drugs.

## 3. Results and Discussion

### 3.1. Core Herb Networks from Herbal Prescriptions

343 herbs were uniquely identified from all 871 prescriptions. By DMIM scoring [6], we obtained frequently used herbs as well as their combinations from all available prescriptions, with Ji Xue Teng as the most frequently used herb. The top 15 herbs in Mr. Li's anti-RA herbal prescription are shown in Table 1. We can see that although the prescriptions are written out individually to patients, the commonly used herbs with different properties are considered to be the most representative of the available herbal prescriptions. Therefore, to capture the common rule of herbal prescriptions, here we focused on the most frequent herbs for the subsequent network analysis. Then we conducted the herb network analysis for these herbal prescriptions. A node in herb network refers to an herb, and an edge connecting two nodes refers to the two significant cooccurrent herbs in herbal prescriptions. By using DMIM score to evaluate the significance of the edge, we constructed two types of the core herb networks, namely, the high-frequency herb network and the intersection herb network for Mr. Li's 871 prescriptions.

For the high-frequency network, only the top frequent herbs were treated as nodes and the cooccurrence herbs in herbal prescriptions identified by DMIM were treated as edges. For example, we found that the top 10 frequent herbs with 45 edges are closely interrelated with each other, forming almost all connected graphs. Among them, Ji Xue Teng and Huang Qi (DMIM score = 18.627), Huo Xue Teng and Ji Xue Teng (DMIM score = 16.517) have the highest DMIM scores. This result suggests that the top 10 herbs are always prescribed in pairs in Mr. Li's practice for RA treatment.

**Figure 2:**
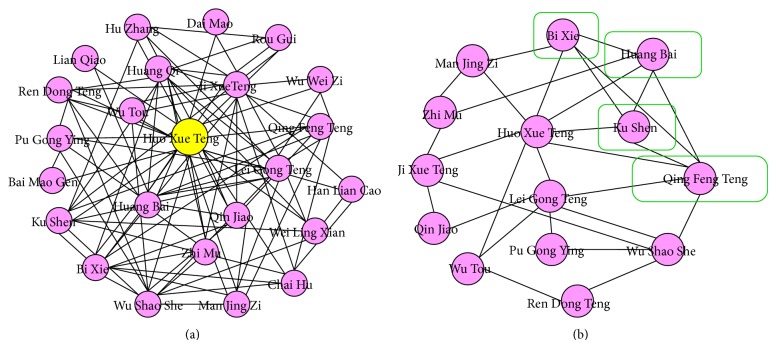
The core intersection herb networks constructed from Mr. Ji-Ren Li's anti-RA herbal prescriptions. (a) The herb subnetworks around each top frequent herb, for example, Huo Xue Teng (*Sargentodoxa cuneata*, the top 2 herb). (b) The intersection herb network created from the intersection herb networks of the top ten frequent herbs. Four herbs circled by the green line are a complete formula of* Qing-Luo-Yin*.

For the intersection herb network, we built every subnetwork by DMIM for each of the top frequent herbs and then intersected the subnetworks, to construct a core network. For example, Figure 2(a) shows the subnetwork around the top 2 herb, Huo Xue Teng (*Sargentodoxa cuneata*). The top ten frequent herb subnetworks contained 21 to 35 nodes and 100 to 200 edges, and the resulted core intersection network has 14 nodes and 29 edges (Figure 2(b)). Interestingly, a complete formula of Mr. Li,* Qing-Luo-Yin* [17], is present in this network.

Herbs and herb combinations present in these two types of herb networks may play a central role in Mr. Li's herbal prescriptions for the treatment of RA.

### 3.2. Potential Synergistic Activities Underlying Core Herb Networks

**Figure 3:**
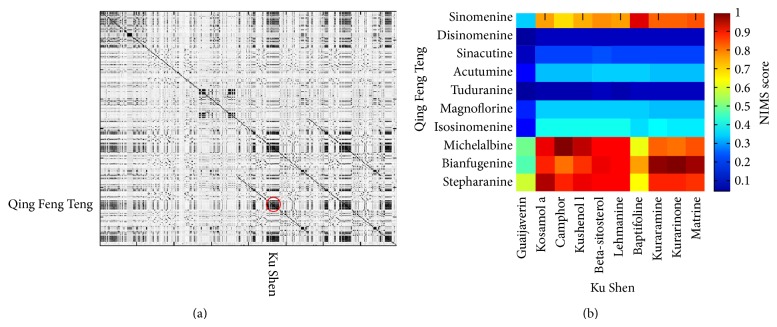
Calculation of synergistic activity among compounds collected from the core herb networks (a). A part of the synergistic module from compounds of Qing Feng Teng (*Caulis Sinomenii*) and Ku Shen (*Radix Sophorae flavescentis*) (b). The horizontal and vertical axes show the compounds of herbs and the intervals of axis represent herbs. The dark areas illustrate a modular feature and indicate strong synergistic interactions of herbs with high synergistic scores calculated by the NIMS method.

Taking the core herb networks as examples, we further performed the network target analysis to computationally determine whether the DMIM extracted herb networks displayed synergistic effects. In this step, we collected available compounds for the core herb networks identified from Mr. Li's prescriptions. The target profile of each compound was predicted by the drugCIPHER method [8]. Then we selected top 100 targets of compounds assembled in each herb to analyze the synergistic activity by evaluating the target interactions in the protein-protein interaction network. When drugCIPHER [8] and NIMS [7] (see Section 2) are applied to the herb networks, we can obviously detect the modular property in the molecular level that denotes the synergistic relationship in the core herb networks (Figure 3(a)). From the outputs of the NIMS score, we found that, in an herb network, not all herbs interact strongly. Instead, only certain herbs or even certain herbal compounds produce clear synergistic effects. For example, in the high-frequency herb network, Ji Xue Teng and Huo Xue Teng (average NIMS score = 0.84), Ku Shen and Qing Feng Teng (average NIMS score = 0.57), Ku Shen and Ji Xue Teng (average NIMS score = 0.4), and Huo Xue Teng and Qing Feng Teng (average NIMS score = 0.31), each of the two herbs has a relatively high NIMS score and tends to produce synergistic activity in different levels. In the intersection herb network, the main constituent of TCM herbal formulae,* Qing-Luo-Yin* (QLY), also has a good NIMS score. A part of the synergistic module from Qing Feng Teng and Ku Shen was shown in Figure 3(b). This figure further illustrated that different compounds in an herb pair obtained diverse synergistic scores. Among them, the combination of compounds such as Sinomenine and Matrine achieves a relatively high score, which agrees with the previously experimental results on the synergistic effects in QLY [17]. Thus, we demonstrate here that the synergistic effects of herb components may be contributed to the modular property of the herb network as well as herbal prescriptions.

### 3.3. Biological Associations between Herb Targets and RA Genes

**Figure 4:**
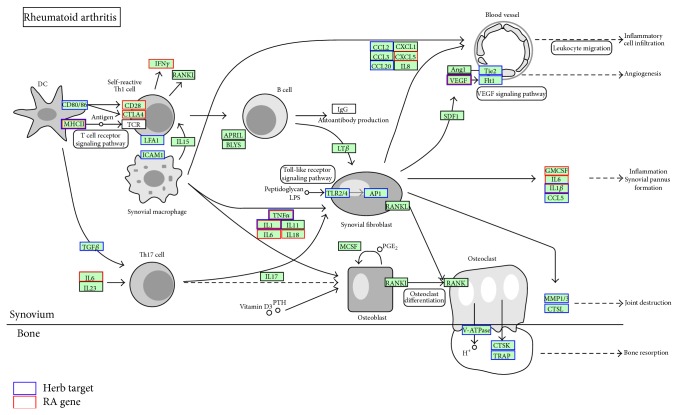
Network association of RA genes and top 15 frequent herbs' targets in the enriched KEGG “rheumatoid arthritis” pathway (KEGG:05323).

Since the targets of each compound of herbs are available by drugCIPHER, we further verify the effectiveness of the top 15 frequent herbs in Mr. Li's prescriptions (Table 1) by evaluating the herbal compounds' targets and their relationship with RA genes in terms of the GO biological processes and pathways. As shown in Table 2, there are RA-related GO terms resulting from targets of the top 15 herbs, which were also enriched with 129 RA-related genes form the CTD database. For example, the overlapped GO terms which resulted from both herb targets and RA genes include angiogenesis [18], cytokine production [19], inflammatory response [20], lymphocyte activation [21], and NF-*κ*B transcription factor activity [22]. The overlapped GO terms also include “defense response to bacterium,” which is worthy of further study. Note that other nonoverlapping GO terms associated with herbs such as the steroid hormone receptor signaling pathway also contributed a lot to the progress of RA [23]. Moreover, by pathway enrichment analysis, we further found that both the RA genes and herb targets are significantly enriched in the pathway of “rheumatoid arthritis” (KEGG:05323) (corrected *P* = 1.55*e* − 15 for RA genes and *P* = 2.83*e* − 12 for herb targets, resp.).  Figure 4 shows the details of the network associations of RA genes and herb targets in the KEGG “rheumatoid arthritis” pathway, providing additional evidence for the anti-RA effects of Mr. Li's herbal prescriptions. These results suggest that the top 15 frequent herbs have a potential to treat RA patients by intervening in the critical biological processes and pathway of RA, which in turn gives evidence for the rationality of the commonly prescribed herbs in Mr. Li's clinical practice.

### 3.4. Network Connections of Anti-RA Herbs and Drugs

**Figure 5:**
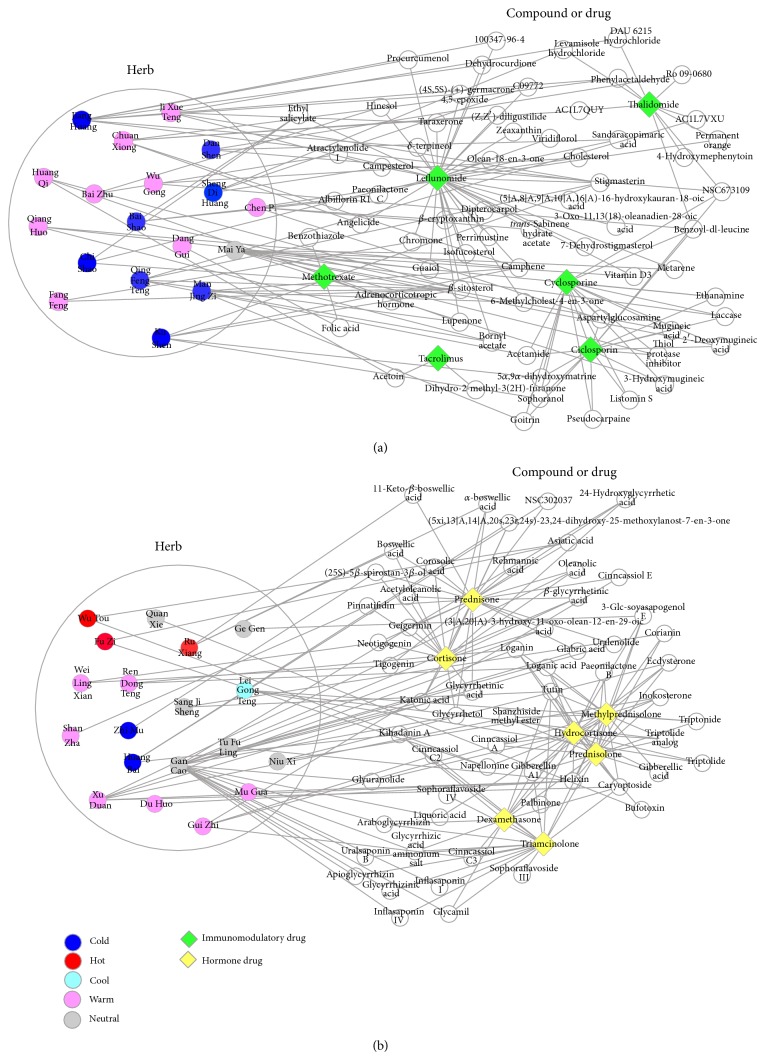
Possible connection between RA herbs and drugs in the networks consisting of herbs, herbal compounds, immunomodulatory drugs (a), and hormones (b).

In TCM, RA patients can be categorized to different syndromes (*ZHENG* in Mandarin), for example, Cold Syndrome and Hot Syndrome. Accordingly, as shown in Table 1, Cold, Hot, Warm, Cool, and Neutral are the basic properties of herbs reflecting the traditional characteristic of TCM. Typically, TCM doctors always use Cool or Cold herbs to treat patients with Hot Syndrome, whereas they use Warm or Hot herbs to treat patients with Cold Syndrome. Cold-tendency and Hot-tendency herbs may be combined to treat RA patients with mixed Hot and Cold Syndromes. On the other hand, in modern medicine, immunomodulatory drugs such as methotrexate and leflunomide [24, 25] and hormones such as glucocorticoids (dexamethasone) [26, 27] are two categories of medicines for the treatment of patients with rheumatoid arthritis [28]. Both categories of medicines can improve the quality of life and delay the disease progression of RA patients via independent but interactive mechanism of actions. By calculating compound networks from the top 50 frequent herbs in Mr. Li's prescriptions, we obtained the herbs that contain ingredients similar to RA immunomodulatory drugs and hormones, respectively. As shown in Figure 5, many herbs contain compounds with targets connected to the selected anti-RA drugs in the network and may exert similar actions on RA. For example, 9 out of top 10 herbs with different properties such as Cold and Hot (Table 1) have compounds that are closely clustered with RA immune and hormone drugs in the network. These results are meaningful since recent studies indicated that the metabolism and immune imbalance are closely associated with patients suffering from Cold Syndrome and Hot Syndrome [29, 30] in many diseases including rheumatoid arthritis [31, 32]. Interestingly, it is noticed that the Cold herbs tend to be enriched in the immune-herb cluster, Hot herbs tend to be enriched in hormone-herb cluster, and Warm herbs are enriched in both clusters. Moreover, compared with the hormone-herb cluster (Figure 5(b)), the immune-herb cluster is less diverse and only contains Cold and Warm herbs, suggesting these herbs tend to exert immunosuppressive or immunoenhancement actions (Figure 5(a)). Although the results are preliminary, the potential connection as well as combinational effects between herbs and drugs is of great importance and deserves further investigation in larger samples and clinical or experimental studies.

## 4. Conclusions

In summary, we developed a TCM network pharmacology strategy and an Herb-Compound-Target-Disease coherent network analysis framework to study herbal prescriptions, the main form of TCM clinical practice. We extracted two types of core herb networks from 871 anti-RA herbal formulae prescribed by a famous TCM doctor. From the core herb networks, our network-based strategy identified cooccurrence herbs and herb pairs with potential synergistic activities. We also predicted the biological basis of the core herb network by calculating the compound's targets from most frequent herbs. We detected that targets of the commonly prescribed herbs are enriched together with RA genes in critical biological processes and pathway of RA. We also indicated that herbs with different properties in prescriptions may have a potential connection with RA immunomodulatory drugs or hormones in the compound networks. Taken together, these results could help uncover the prescription rules underlying herbal formulae in the clinical practice of TCM practitioners. Moreover, such network-based analyses not only can develop a holistic understanding of herbal remedies but also may facilitate the following pharmacological evaluation of herbal treatments. Clearly, this study is only the first step to learn the common rule of herbal prescriptions. We will continue to conduct in-depth analysis for understanding the scientific basis of herbal prescriptions in our future work. Although more powerful methods, more data on TCM prescriptions, and clinical or experimental evaluations are still required, we believe that this TCM network pharmacology strategy is suitable to study herbal formulae both in the herb level and in the molecular level and thus can serve as a novel approach to further understand the professional experience embedded in TCM prescriptions.

## Figures and Tables

**Table 1:** Top 15 herbs in Mr. Ji-Ren Li's antirheumatoid arthritis herbal prescriptions.

Chinese name	Latin name	Property	Frequency	Normalized frequency
Ji Xue Teng	*Millettia reticulata *Benth	Warm	503	5.10%
Huo Xue Teng	*Sargentodoxa cuneata *	Neutral	499	5.06%
Huang Qi	*Astragalus membranaceus *(Fisch.) Bunge.	Warm	481	4.87%
Dang Gui	*Radix Angelicae sinensis *	Warm	430	4.36%
Quan Xie	*Scorpio *	Neutral	399	4.04%
Qing Feng Teng	*Caulis Sinomenii *	Neutral	396	4.01%
Ku Shen	*Radix Sophorae flavescentis *	Cold	323	3.27%
Wu Tou	*Aconitum carmichaeli *Debx.	Hot	306	3.10%
Wu Gong	*Scolopendra *	Warm	287	2.91%
Huang Bai	*Cortex Phellodendri *	Cold	283	2.87%
Bi Xie	*Rhizoma Dioscoreae Hypoglaucae *	Neutral	280	2.84%
Ren Dong Teng	*Caulis Lonicerae *	Cool	257	2.60%
Qin Jiao	*Radix Gentianae macrophyllae *	Cold	254	2.57%
Wu Shao She	*Zaocys dhumnades *	Neutral	235	2.38%
Pu Gong Ying	*Taraxacum mongolicum *Hand.-Mazz.	Cold	227	2.30%

**Table 2:** Overlapped RA-related GO terms between top 15 herb targets and RA genes.

Category	Enriched GO terms	Corrected *P*-value
Herb targets	Response to wounding	2.38*e* − 127
	Angiogenesis	1.21*e* − 26
	Inflammatory response	8.60*e* − 71
	Regulation of cytokine production	6.12*e* − 26
	Immune response	2.61*e* − 88
	Leukocyte activation	3.41*e* − 38
	Regulation of lymphocyte activation	4.52*e* − 23
	Defense response to bacterium	3.56*e* − 10
	Positive regulation of NF-*κ*B transcription factor activity	6.04*e* − 12

RA genes	Response to wounding	1.48*e* − 15
	Angiogenesis	5.79*e* − 6
	Inflammatory response	5.76*e* − 26
	Regulation of cytokine production	1.65*e* − 26
	Immune response	6.29*e* − 39
	Leukocyte activation	7.28*e* − 30
	Regulation of lymphocyte activation	1.25*e* − 31
	Defense response to bacterium	7.27*e* − 12
	Positive regulation of NF-*κ*B transcription factor activity	2.54*e* − 6

## Abbreviations

DMIM: Distance-based mutual information model
drugCIPHER: Drug-target prediction by correlating protein interaction network and phenotype network
GO: Gene ontology
MI: Mutual information
NIMS: Network-based identification of multicomponent synergy
RA: Rheumatoid arthritis
PPI: Protein-protein interaction
QLY: Qing-Luo-Yin
TCM: Traditional Chinese Medicine.
